# Fluorescent Probe for the pH-Independent Rapid and Sensitive Direct Detection of Urease-Producing Bacteria

**DOI:** 10.1021/acs.analchem.4c05182

**Published:** 2024-12-16

**Authors:** Werner C. Albrich, Christian R. Kahlert, Susanne Nigg, Luciano F. Boesel, Giorgia Giovannini

**Affiliations:** † Division of Infectious Diseases, Infection Prevention and Travel Medicine, Kantonsspital St. Gallen, Kantonsspital St. Gallen, Rorschacher Strasse 95, St. Gallen 9007, Switzerland; ‡ Infectious Diseases and Hospital Epidemiology, Children’s Hospital St. Gallen, Claudiusstr. 6, St. Gallen 9006, Switzerland; § Empa, Swiss Federal Laboratories for Materials Science and Technology, Laboratory for Biomimetic Membranes and Textiles, Lerchenfeldstrasse 5, St. Gallen 9014, Switzerland

## Abstract

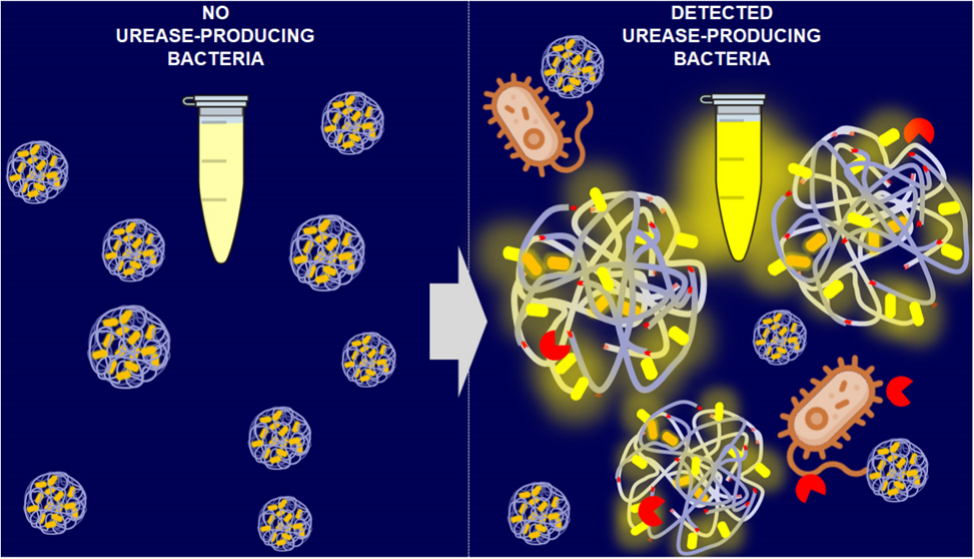

Urease-producing bacteria are highly relevant in medicine due to their role in various pathogenic processes and their impact on human health, causing serious medical conditions such as peptic ulcer disease, gastric cancer, and respiratory and urinary tract infections. In this work, we designed fluorescent polymeric particles (PNP_FITC) to enable the detection of urease-producing bacteria by targeting the enzymatic activity of urease. In particular, the PNP_FITC matrix is degraded by urease, leading to a measurable increase in the intensity of the fluorescent signal. This approach is designed to directly sense urease activity and is therefore not affected by environmental parameters, unlike standard methods based on the quantification of enzymatic metabolites (i.e., NH_3_ and CO_2_). PNP_FITC exhibited a linear response in the urease range of 0–7.5 U/mL, with a calculated limit of detection of 0.4 U/mL. The direct detection of enzymatic activity makes PNP_FITC suitable for detecting urease-producing bacteria (*Klebsiella pneumoniae* and *Enterobacter cloacae*) with a detection limit of 10 ∧ 3 bacteria/mL, which were not detectable using the pH-based method employed as the reference in this work. Given the improvements achieved with PNP_FITC in terms of robustness, sensitivity, and selectivity of urease detection compared to the standard methods, this approach represents a step forward toward the development of advanced point-of-care, enabling the prompt diagnosis of bacterial infections.

## Introduction

Urease is an enzyme synthesized by a large variety of organisms, including plants, bacteria, and fungi. It catalyzes the hydrolysis of urea into ammonia (NH_3_) and carbon dioxide (CO_2_). The determination of urease activity is extremely important in several areas, such as agriculture,^1^ environmental science,^2^ and medicine.^3^ Urease is considered a virulence factor of pathogenic bacteria since it is involved in a series of processes that allow bacteria to colonize and induce a strong host inflammatory response.^4,5^ The hydrolysis of urea into NH_3_ neutralizes the acid environments favoring bacterial proliferation.^6^ Moreover, it is involved in the delivery of toxins to the host,^7^ contributes to inflammation, and promotes biofilm formation.^8^ The detection of urease is crucial in medicine, as it enables the diagnosis of infections caused by pathogenic urease-producing bacteria, such as *Helicobacter pylori*, which produces urease to survive the acidic environment of the stomach, leading to peptic ulcers and gastric cancer. Similarly, *Proteus spp*. and *Klebsiella spp*. are associated with kidney stones^9^ and bladder diseases.^10,11^ Additionally, detecting urease-producing bacteria could provide a novel method for diagnosing pneumonia, particularly in cases involving *Klebsiella pneumoniae*, a significant pathogen in hospital-acquired pneumonia^12^ that releases urease due to its nitrogen metabolism.^13^ Ventilator-associated pneumonia (VAP) is a severe nosocomial infection that occurs in mechanically ventilated patients in intensive care units. It is one of the most frequent life-threatening infections in a clinical setting, affecting up to 27% of patients in intensive care. VAP is often associated with significant pathogens such as *K. pneumoniae*, *Pseudomonas aeruginosa*, *Enterobacter spp*., *Escherichia coli*, and *Staphylococcus aureus*.^14^ The expression of urease in VAP-associated pathogens is notably higher compared to community-acquired pneumonia, which is typically caused by *Streptococcus pneumoniae* or *Haemophilus influenzae*.^15^ While urease detection is not currently the primary method for diagnosing pneumonia, it can assist in identifying *Klebsiella spp*., thereby supporting more accurate diagnosis and treatment.^16,17^ Additionally, understanding urease production in these bacteria could provide insights into its role in infection, potentially leading to the development of targeted therapies and preventive measures.^18−21^

Urease tests have been developed to identify pathogens in human samples, either in vitro or in vivo, by measuring the products of the enzymatic activity. These tests are based on two main approaches: (i) pH-based detection of NH_3_, which causes alkalization of the surrounding environment (Christensen urea agar method and the rapid urease test-RUT) and (ii) analysis of CO_2_ released as a gas, which is detected using breath sensors. The primary analytical techniques employed for urease breath tests (UBTs) are infrared spectroscopy and mass spectrometry, which allow for the quantification of exhaled CO_2_.^22,23^ Despite being the first choice for the diagnosis of *H. pylori* due to its limited invasiveness, UBT is subject to a high risk of interference.^24^ Moreover, infrared and mass spectrometry require trained personnel for both measurement and data analysis.^25^ Affordable and user-friendly alternative methods for detecting urease-producing bacteria rely on color changes or variations in fluorescent signals.^26,27^ However, these methods have notable disadvantages. The pH-based methods are affected by nonurease-related pH changes, which limits their applicability to nonhuman samples under controlled conditions.^28,29^ Additionally, they are time-consuming because bacteria must be cultivated for at least 24 h and often require the addition of urea to induce enzymatic activity.^30,31^ In contrast, breath-based methods face significant challenges, such as the labor-intensive process of sample collection.^32,33^ Moreover, various sources of CO_2_, including other bacterial strains, food, and medications, can lead to false positives when detecting *H. pylori*.^34,35^ Therefore, there is a critical need for improved methods for detecting urease-producing bacteria to enable rapid and accurate diagnosis of infections.

In this work, we present a rapid and affordable fluorescence-based probe designed for the rapid detection of urease activity. The fluorescent signal of the probe is not affected by the intrinsic color of human fluids, making it suitable for detecting urease-producing bacteria in expectorate, urine, and blood.^36^ Due to its sensitivity and robustness, the proposed probe could pave the way for the implementation of point-of-care diagnostics, enabling the prompt administration of the appropriate treatment. This is particularly important for managing serious infections, such as *H. pylori*-induced peptic ulcers and nosocomial pneumonia caused by *K. pneumoniae*.

## Experimental Section

Tris(2-aminoethyl)amine (TAEA), fluorescein isothiocyanate isomer I ≥ 97.5% (FITC), isoprene diisocyanate 98%, a mixture of isomers (IPDI), ethanol, tetrahydrofuran (THF), fluorescein sodium salt, urea BioReagent recombinant, β-glucosidase from almonds, proteinase from Aspergillus melleus, glucose oxidase from *Aspergillus niger*, lactate oxidase from Aerococcus viridans, LB-broth (Leenox), NaCl, KCl, Na_2_HPO_4_, KH_2_PO_4_, NaOH, and HCl were purchased from Sigma-Aldrich. Phosphate-buffered saline 10 mM pH7.4 (hereafter referred to as “PBS”) was prepared by solubilizing salts at different concentrations NaCl 0.137 M, KCl 0.0027 M, Na_2_HPO_4_ 0.01 M, KH_2_PO_4_ 0.0018 M in deionized (DI) water. The desired pH was reached by adding 1 M NaOH or 1 M HCl. Urease (recombinant, Abbexa) was purchased from LubioScience GmbH. Bacteria were isolated from human samples (urine and wound exudate) collected from hospitalized patients at the Kantonsspital St. Gallen and stored at −80 °C in glycerol solution.

### Synthesis of PNP_FITC: FITC

(3.15 mg, 0.01 mol) was dissolved in 2 mL of dry THF, and it was sonicated until completely solubilized. The solution was flushed with nitrogen, and subsequently, TAEA (150 μL, 1 mol) was added under nitrogen and continuous stirring. The solution was diluted to 20 mL with dry THF and stirred under nitrogen in the dark for 1 h. IPDI (430 μL, 2 mol) was diluted with dry THF (1.57 mL) to reach 2 mL as the final volume. This solution was added dropwise to the FITC-TAEA solution during sonication (10% amplitude). The solution was kept in an ice bath during sonication to prevent an increase in the temperature. The so-achieved solution was stirred for an additional 1 h in the dark and then added dropwise in DI water (50 mL) during sonication to induce the formation of the particle. The so-achieved suspension was first centrifuged at 425 g for 8 min to isolate larger particles. The supernatant was further centrifuged at 10 621 g for 8 min to isolate the particles from small components and unfolded polymeric material. The supernatant was discharged, whereas the pellet isolated at this stage was resuspended in DI water by sonication (10% amplitude, 30 s). The so-achieved PNP_FITC was stored at a concentration of 1 mg/mL in DI water at 4 °C in dark conditions. All experiments were accomplished using two batches over almost 1 year, proving the stability of the probe over time.

### Dynamic Light Scattering Analysis

The morphology of PNP_FITC was monitored by a Zetasizer NanoZS (Malvern Instruments Ltd., Malvern, UK) through a HeNe laser of 633 nm with a backscatter angle of 173°. PNP_FITC was diluted to a final concentration of 20 μg/mL in DI water. 1.5 mL of disposable plastic cuvettes were used to measure size and polydispersity index (PDI). The parameters applied in the measurements were room temperature (∼25 °C); dispersant: viscosity of 0.8872 cP, reflective index of 1.330; material: absorption 0.01, refractive index of 1.59. The final average values were reported through three measurements.

### Confocal Microscope Analysis

This work used a laser scanning confocal microscope (LSM 780, Zeiss) LASOS Ar-lon Laser (Zeiss, Model LGN 3001, Remote Control RMC 7812 Z2, 490 nm).

### Transmission Electron Microscopy Analysis

Images were taken on a JEOL 2200FS TEM instrument equipped with an in-column Omega-type energy filter (Joel). In total, 5 μL of the sample was dropped on “Carbon Films on 200 Mesh Grids Copper” and allowed to evaporate.

### Fourier Transform Infrared Spectroscopy

Spectra were recorded with a Varian 640-IR, software Agilent Resolutions PRO on dried untreated and urease-treated samples purified from the protein by centrifugation (10 621 g, 8 min).

### Urease Assay

Both PNP_FITC and urease samples were diluted with PBS at defined concentrations and were added in equal volume (60 μL) to a 96-well plate, reaching the desired concentrations. The signal of the fluorescent probe was monitored with a microplate reader (BioTek Synergy H1, Instruments Inc., USA) with the following parameters: excitation wavelength 470 nm, emission wavelength 530 nm, room temperature, and automatic reading every 10 min. LOD was calculated with the formula: 3.3δ/slope, where δ is the standard deviation of the blank. The *V*_max_ and *K*_m_ of the Michaelis–Menten equation were obtained by using GraphPad.

### Bacterial Assay

LB-broth was prepared by solubilizing the powder in deionized water at a concentration of 20 g/L and autoclaved at 121 °C for 15 min. 120 μL of the isolated bacteria stored in a glycerol solution was cultured in fresh LB-broth (6 mL) overnight under shaking at 37 °C. Stock solutions of 1 × 10 ∧ 8 bacteria/mL bacteria were prepared, diluting the overnight culture with LB-broth until the optical density of the sample was equal to 0.3 au. The tested bacterial samples were prepared by diluting the LB-stock solution in PBS at defined concentrations. PNP_FITC and bacterial samples were diluted to the desired concentration in PBS and added in equal volumes (60 μL) to a 96-well plate, reaching the desired concentrations. The signal of the fluorescent probe was monitored with a microplate reader (Infinite M Plex, Tecan AG, Männedorf, Switzerland) with the following parameters: excitation wavelength 470 nm, emission wavelength 530 nm, room temperature, and automatic reading every 10 min. LOD was calculated with the formula: 3.3δ/slope, where δ is the standard deviation of the blank.

### pH-Based Method Assay

The reagent solution was prepared and tested as reported in the literature.^37^ For the urease and bacterial assay, 190 μL of the reagent solution was added to the 96-well plate, with the addition of 10 μL of enzyme/bacterial solution reaching the desired concentrations. The signal of the reagent solution was monitored with microplate readers using the following parameters: excitation wavelength 490 nm, emission wavelength 520 nm, room temperature, and automatic reading every 10 min.

### Statistical Analysis

Statistical analysis was accomplished using Origin 2020b software. To compare different samples, one-way ANOVA with a post hoc test was used to define the relationship between groups (*p* < 0.05, Tukey test, Levene’s test, actual power).

## Results and Discussion

**Figure 1:**
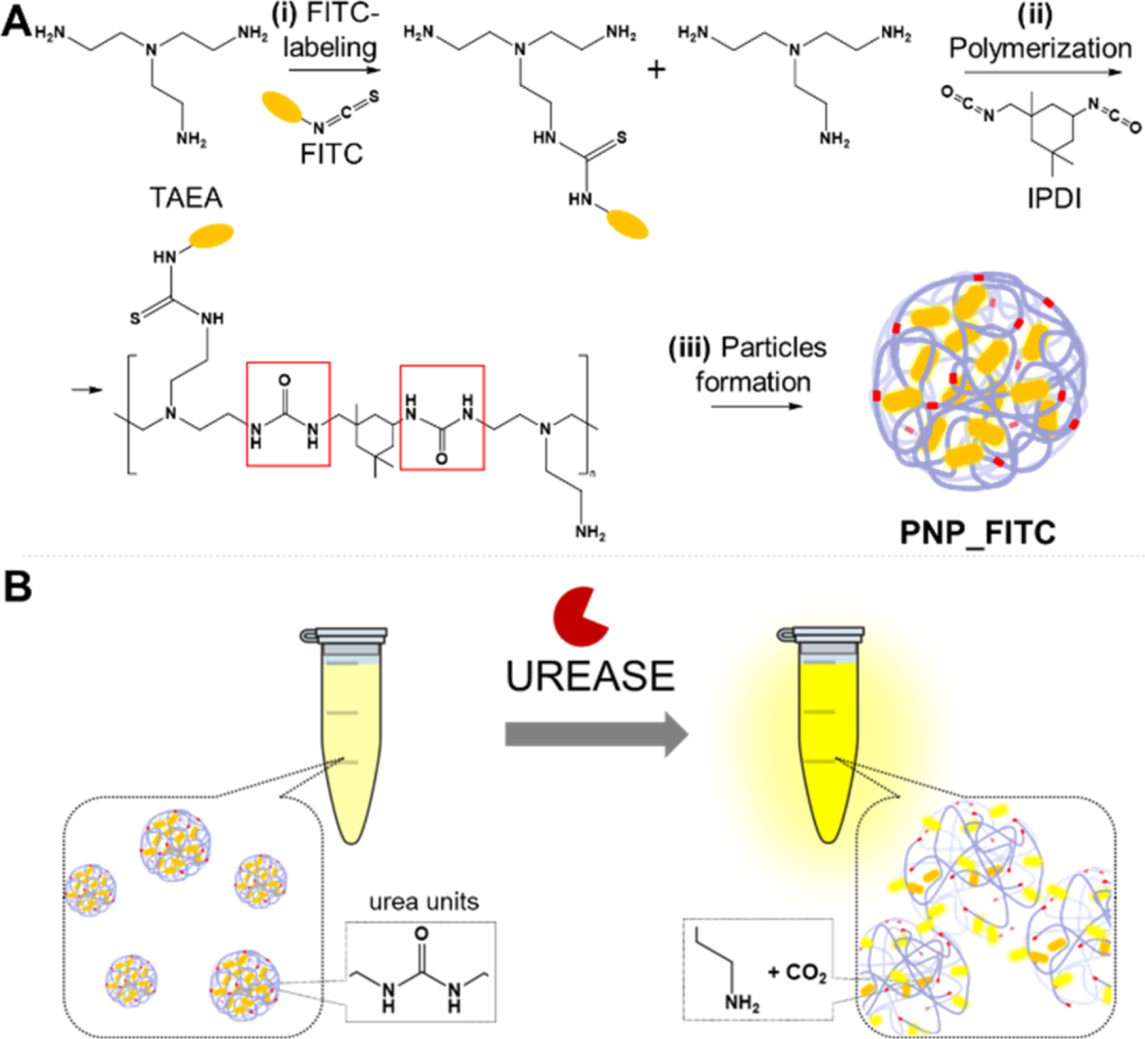
Scheme of the synthetic procedure for PNP_FITC (A) and the mechanism of the fluorescent-based direct urease detection (B).

We designed a fluorescent-based detection approach for urease-producing bacteria targeting directly the enzymatic activity rather than its products (i.e., NH_3_ and CO_2_),^38^ aiming to improve the sensitivity and selectivity of detection. In particular, we synthesized polymeric particles with two main features: (i) the presence of urease-cleavable units and (ii) a high rate of fluorescent labeling of the polymeric matrix. The scheme in Figure 1A shows the synthetic procedure used to synthesize the fluorescent probe, named PNP_FITC. First, we labeled tris(2-aminoethyl)amine (TAEA) with fluorescein isothiocyanate (FITC) using 100:1 as the molar ratio (Figure 1A-i). Such a functionalization degree was chosen to achieve a suitable concentration of dye in the final polymeric matrix, enabling a reversible self-quenching of the fluorescent signal. Subsequently, we treated the labeled TAEA with isoprene diisocyanate (IPDI) to induce the polymerization by the reaction of the remaining amino groups of TAEA with the isocyanate groups of IPDI (Figure 1A-ii). This step leads to the formation of urea units in the polymeric matrix. The so-achieved THF-based polymer solution was added dropwise in deionized water to form the polymeric particles by the precipitation method (Figure 1A-iii). After sonication, the so-obtained PNP_FITC particles were purified by centrifugation and resuspended in water. The direct mechanism of the detection of urease is schematized in Figure 1B. The high concentration of FITC in the probe achieved with the selected TAEA-FITC ratio caused interactions among adjacent molecules leading to the self-quenching phenomenon^39,40^ due to which PNP_FITC exhibited a low fluorescent intensity. However, when the polymeric matrix was cleaved by urease, the expansion of the polymeric matrix and the progressive separation of FITC molecules led to a remarkable increase in the fluorescent signal of the PNP_FITC.^41^ Given the small dimension of the monomers forming the matrix (TAEA and IPDI), an increase in the functionalization rate of TAEA (e.g., 10:1) would have (i) limited reversibility of the self-quenching phenomenon, (ii) limited polymerization due to the lower amount of amino groups available to react with the isocyanate-derivative monomer, and consequently (iii) fewer urease-cleavable units in the matrix.

**Figure 2:**
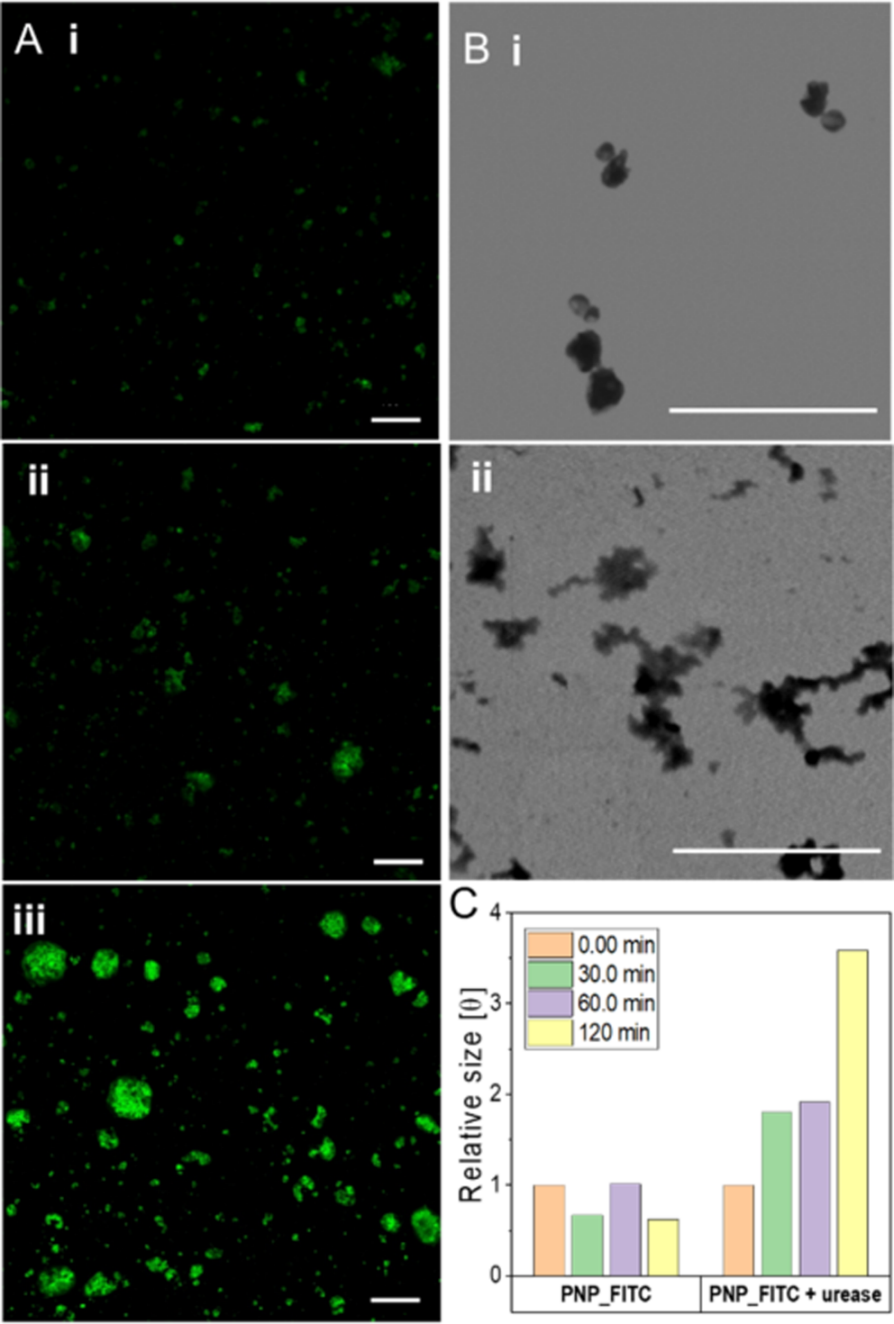
(A) Confocal analysis showing the limited size and fluorescence of the untreated probe (i) and the changes upon urease treatment (15 U/mL) over time (ii-30 min; iii-60 min). Scale bar = 100 μm. (B) TEM micrographs confirmed the matrix expansion of PNP_FITC after urease treatment (ii-15 U/mL) compared to the untreated sample (i). Scale bar 3 μm. (C) DLS results showed the increase of particle size over time when treated with urease (15 U/mL), as well as the stability of the untreated sample.

Figure S1A–C shows the optical properties of PNP_FITC when treated with urease at different concentrations. 470 and 530 nm were selected as the excitation and emission wavelengths, respectively. The large Stoke’s shift (60 nm) limited the interference of the excitation with the detected emission. The envisioned mechanism of detection of the probe was confirmed experimentally by measuring the optical properties of the particles treated with urease and also evaluating the particle’s structure over time by DLS, TEM, and confocal microscopy. Both confocal and TEM analyses enabled us to observe the structural changes of the matrix when treated with urease. As shown in Figure 2A, the size and fluorescence of PNP_FITC were limited when suspended in pure water (i) but remarkably increased over time once treated with urease (15 U/mL) after 30 (ii) and 60 (iii) minutes. TEM analysis (Figure 2B) showed that in pure water, PNP_FITC was spherically shaped (i) but after urease treatment the enzymatic cleavage of the urea units in the polymeric matrix led to the random expansion of the particles (ii). DLS analysis allowed tracking the size change of PNP_FITC over time, highlighting that, whereas the diameter of PNP_FITC suspended in water did not vary significantly, a 4-fold increase of size was observed after 120 min of urease addition (Figure 2C). The cleavage of the polymeric matrix upon urease treatment was confirmed by Fourier transformation infrared (FT-IR) spectroscopy, showing the increased intensity of the peaks assigned to the amine and carbonyl groups in the treated samples compared to those in the untreated one (Figure S1E).^42^

The efficacy of the PNP_FITC in detecting urease was evaluated by measuring the variation of the fluorescent signal when treated with different concentrations of the enzyme by a specifically designed 96-well assay described in detail in the Experimental Section. 100 μg/mL of PNP_FITC was selected as the optimal concentration of the probe given the linear increase of the fluorescent signal with the increase in urease compared to the other concentrations tested (i.e., 500, 250, and 50 μg/mL) (Figure S1D). The limited responsiveness of the assay observed when the probe was used at higher concentrations (500 and 250 μg/mL) was attributed to the proximity between particles causing quenching of the fluorescent signal between FITC molecules belonging to neighboring probes, preventing the increase of the fluorescent signal. On the other hand, at the lower concentration (50 μg/mL), the limited amount of PNP_FITC available limited the variation of the signal and thus the responsiveness of the probe.

**Figure 3:**
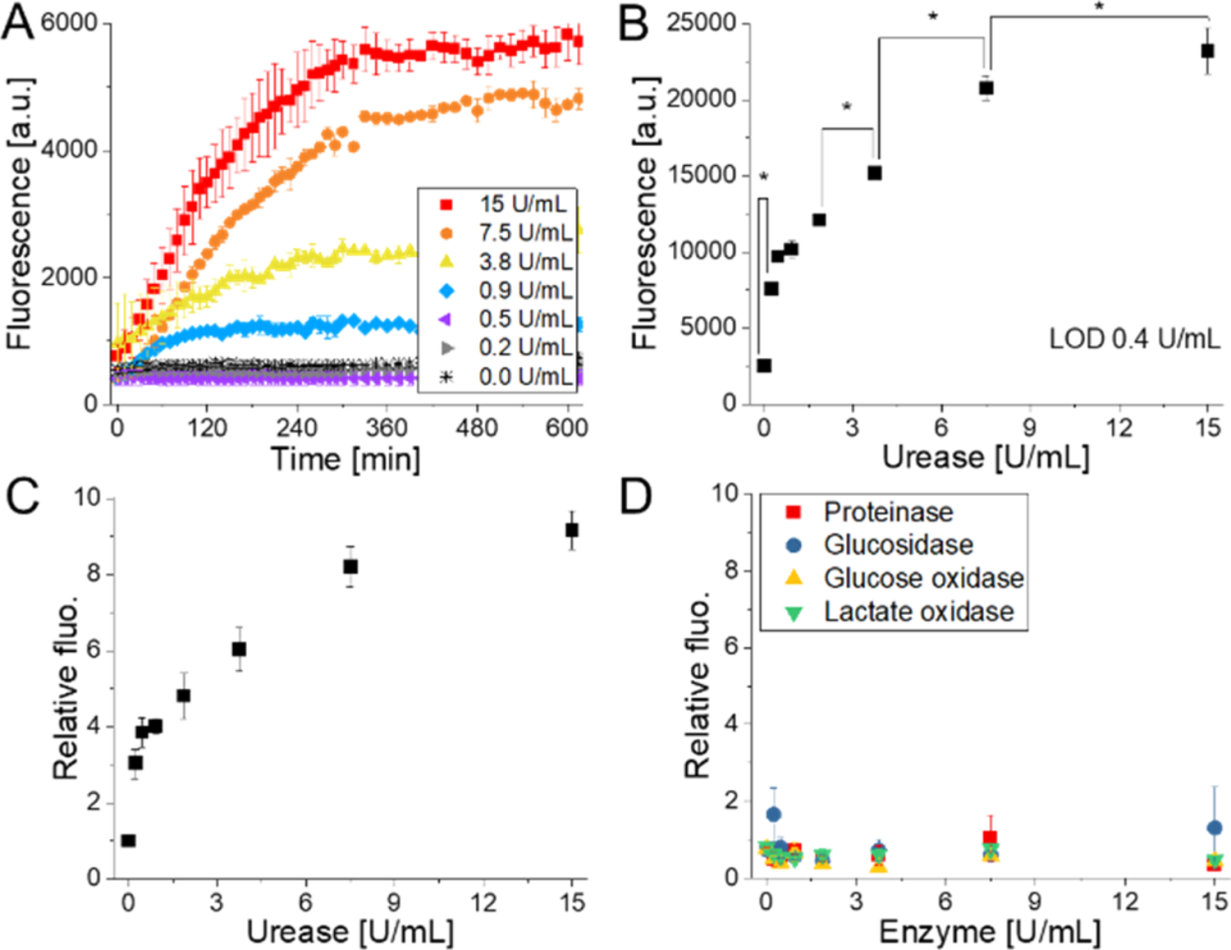
(A) PNP_FITC’s signal increased over time upon urease treatment. (B) The intensity increased linearly with the concentration of urease used (*R*^2^ = 0.92), leading to an LOD of 0.4 U/mL. *Means are significantly different from each other. Statistical analysis ANOVA one-way, *p* < 0.05, Tukey test, *n* = 3. (C) Variation of the relative fluorescence intensity (*I*_t300_/*I*_t0_) with the increase of urease concentration. The values are reported as the average values ± sd, *n* = 3. (D) Selectivity studies showed that PNP_FITC is stable when treated with physiologically relevant enzymes.

The production of urease is influenced by several factors (e.g., bacterial strain, growth conditions, initial concentrations). It is therefore impossible to foresee the amount of enzyme produced by the bacteria. We chose to test urease in the concentration range of 0–15 U/mL, considering values reported in the literature as the amount of urease produced by bacteria under optimal conditions.^43,44^ After 300 min of urease addition, the PNP_FITC signal was stable at any concentration of urease tested (Figure 3A). Given these results, we selected 5 h as the turnaround time to ensure the reliability of the assay and a robust outcome, although we observed that the signal of the probe reached the plateau within less than 2 h when treated with low urease concentrations. PNP_FITC showed a linear increase of the fluorescent signal with the increase of urease concentration in the range of 0.2–7.5 U/mL (*R*^2^ 0.92) with a calculated limit of detection (LOD) of 0.4 U/mL (Figure 3B). It is reported that the minimum urease activity for *H. pylori*-positive patients is 1.5 U/mL, thus proving the suitability of our method for real application.^45^ PNP_FTIC showed an improved sensitivity compared with commercial standard methods such as the colorimetric NH_3_-based Berthelot assay for which an LOD of 0.75 U/mL is reported. Although the Nessler reagent achieves even greater sensitivity (LOD: 0.02 U/mL), its application is restricted due to reagent toxicity.^46^ Statistical analysis showed that the values measured down to 1.8 U/mL were significantly different from each other, confirming the robustness of the proposed direct detection method. The significant effect of the urease concentration on the fluorescent signal is further reported in Figure S2. The responsiveness of the probe was pointed out by plotting the relative intensity values, calculated as the ratio between the intensity after 5 h and the initial value, as reported in Figure 3C. The signal variation in response to urease was remarkable, with an increase in the fluorescent signal of 9-fold when treated with the highest concentration of urease tested (15 U/mL). The selectivity of the probe for urease was confirmed by treating PNP_FITC with glucosidase, proteinase, glucose oxidase, and lactate oxidase, which are involved in the metabolic processes of the bacteria. The enzymes were tested at the same concentrations of urease, i.e., from 0 to 15 U/mL. As shown in Figure 3D, the fluorescent signal of PNP_FITC remained stable upon treatment with the selected enzymes, indicating that the matrix of the particles is not affected by hydrolase and oxidase enzymes. These results confirmed the expected selectivity of the probe for the urease activity. The sensitivity between the probe PNP_FITC and the analyte urease was evaluated by comparing parameters determined from the Michaelis–Menten equation (see eq 1).^47^ In this model, the kinetic rate of the enzymatic reaction is evaluated concerning the probe concentration ([*S*]), the substrate required to achieve saturation (*V*_max_), and the Michaelis constant (*K*_m_)
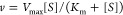


*K*_m_ is one of the key parameters used to indicate the affinity of the enzyme for a substrate; in particular, the higher the *K*_m_, the lower the affinity of the protein for the substrate. *K*_m_ was found to be 3636 μg/mL, suggesting that PNP_FITC can detect lower^48^ or comparable^49^ levels of urease effectively as those reported in the literature, an indication of high sensitivity and efficiency of detection.^50−52^ The high *V*_max_ calculated (107 684 intensity [a.u.]/min) supported the strong signal variation in response to the enzymatic activity, thus the robust detection capability of the approach. Due to the uniqueness of our direct detection mechanism, the *K*_m_ and *V*_max_ values calculated for our probe could not be compared with those found with other approaches proposed in the literature.

**Figure 4:**
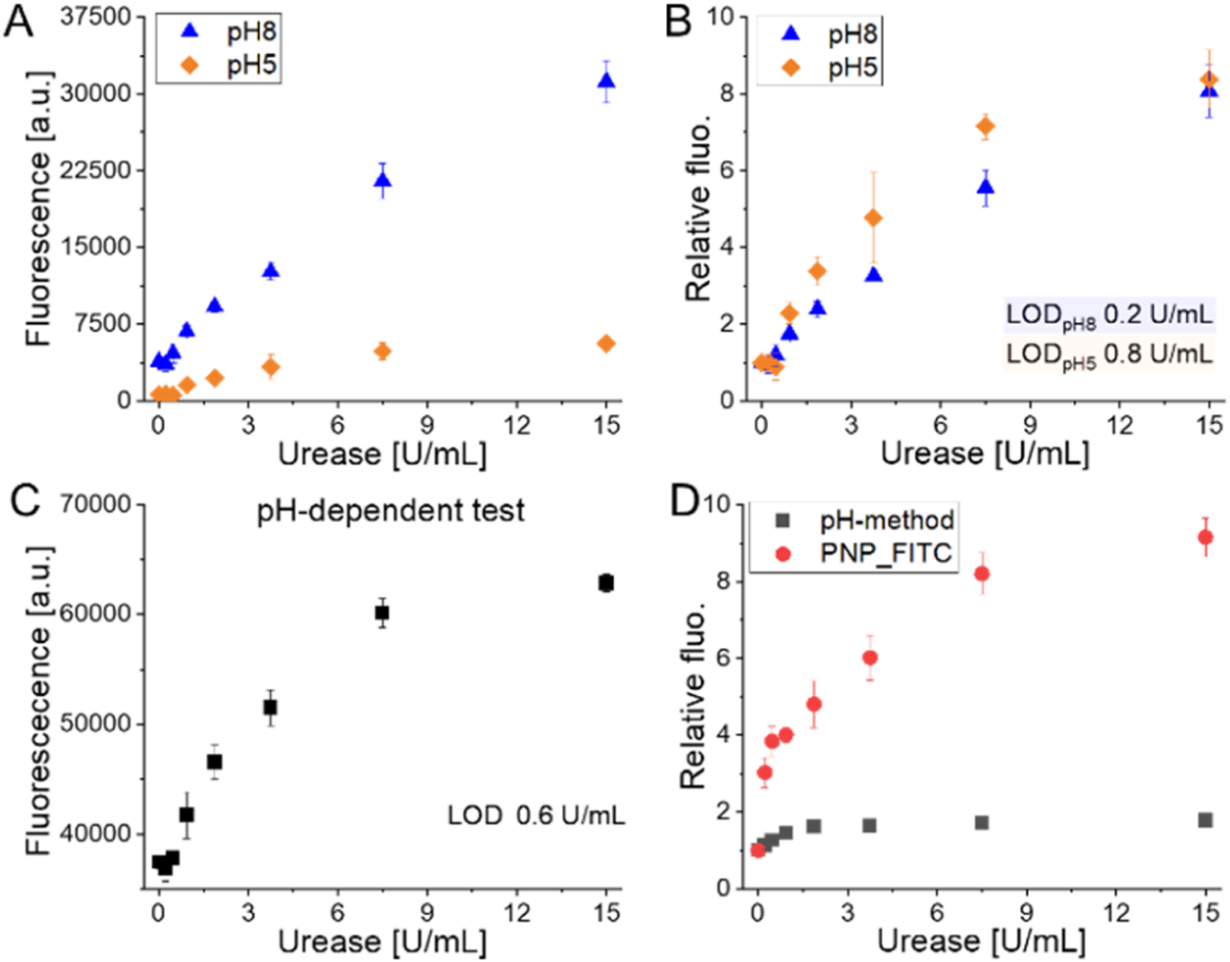
(A) Absolute fluorescence signal of PNP_FITC varies with different concentrations of urease at pH 5 and 8 due to the pH sensitivity of the incorporated dye. (B) The relative value changes are consistent between the two pH values, indicating that the detection mechanism is unaffected by environmental pH. (C) Variation of the fluorescent signal obtained with the pH-based method with a calculated 0.6 U/mL LOD. (D) Variation of the fluorescent signal achieved with the two methods.

One of the advantages envisioned for our direct urease detection is the autonomy concerning physiological pH variation. It is well-known that the environmental pH can become acidic or alkaline when the tissue is colonized by bacteria, depending on the type of infection (i.e., bacterial strains, tissue, nutrient availability). Such pH variation is not directly related to urease activity but can be induced by the accumulation of amines during extracellular matrix degradation or by the accumulation of metabolites, such as lactic acid. Due to these interfering parameters, the pH-based urease approaches can lead to false-positive and false-negative outcomes. To confirm that the proposed probe is not influenced by pH variation, the detection efficacy of PNP_FITC was tested at pH 5 and 8, selected as the two extremes of the biologically relevant pH range. As shown in Figure 4A, the absolute intensity of the PNP_FITC measured at pH 5 was lower compared to the one observed at pH 8 at any concentration of urease tested (from 0 to 15 U/mL) due to the pH sensitivity of FITC. Nevertheless, as reported in Figure 4B, the fluorescent signal varied with the same rate in response to the urease concentration, regardless of the environmental pH, and thus the relative fluorescence increased linearly and with the same rate at the different pH, proving that the detection of urease is not affected by the environmental pH. It is worth mentioning that, despite the well-known pH sensitivity, FITC was selected for the probe synthesis due to (i) its self-quenching properties, (ii) the availability of reactive groups for covalent conjugation to the particle’s matrix, and (iii) its affordability.

To prove the advantage of the proposed direct detection approach, the PNP_FITC was compared with a fluorescent pH-based method reported in the literature and used as the reference in this work thanks to the detailed experimental section that allowed us to replicate the procedure.^37^ As reported in Figure 4C, a linear increase of fluorescence was achieved with the pH-based method in the urease concentration range of 0.2–7.5 U/mL (*R*^2^ = 0.9). Even though the LOD calculated for the pH-based method was comparable with the one achieved with PNP_FITC (0.6 and 0.4 U/mL, respectively), the main difference observed was the rate of signal variation. As reported in Figure 4D, whereas a 9-fold increase of the fluorescent signal was observed treating PNP_FITC with 15 U/mL, the same concentration of enzyme led only to a two-time increase of the signal for the pH-based method. This observation confirmed the improved robustness and reliability of the direct approach proposed with PNP_FITC.

**Figure 5:**
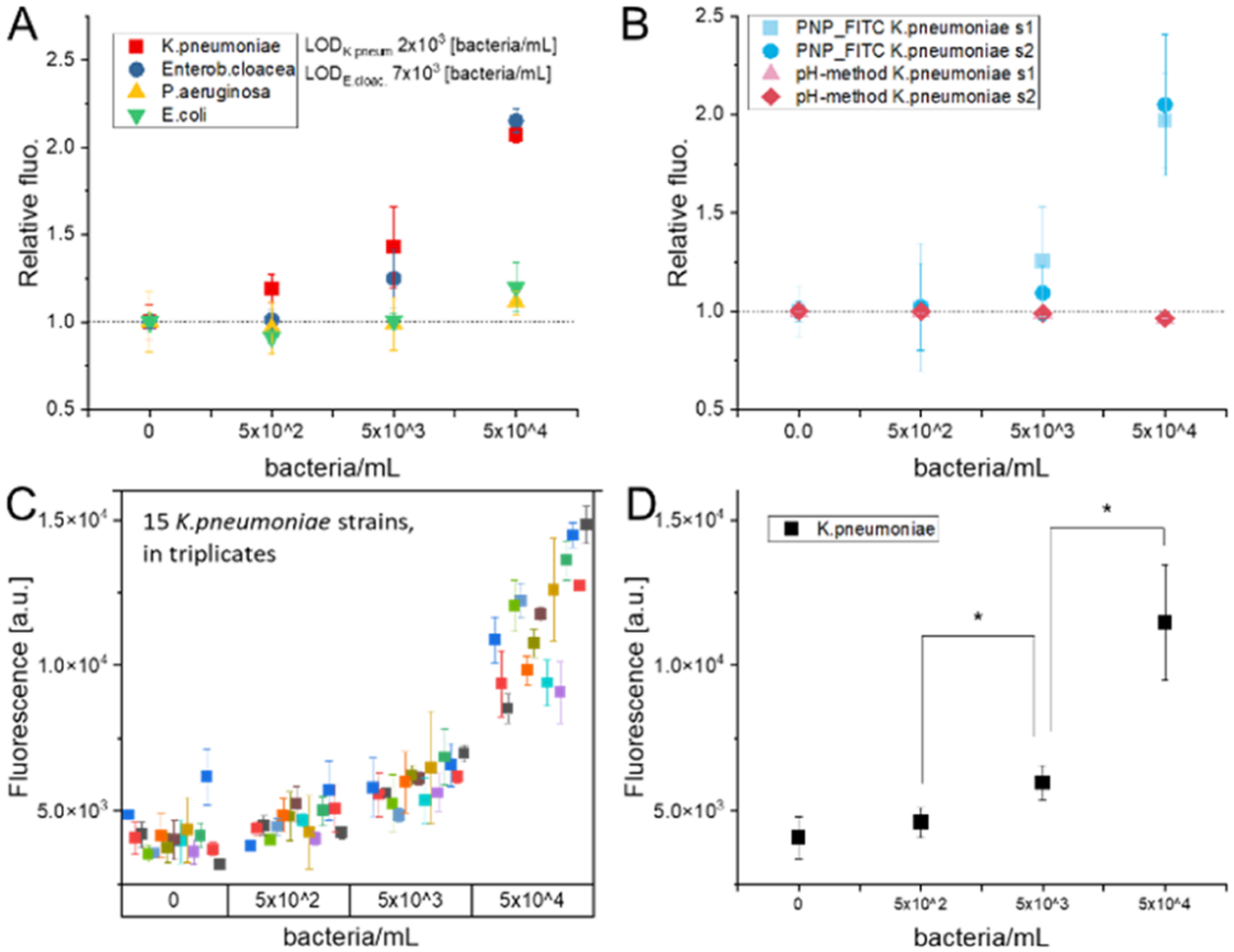
(A) Discrimination among bacteria based on their urease activity using PNP_FITC. (B) Comparison between PNP_FITC and the pH-based method in detecting *K. pneumoniae* strains 1 and 2. (C) Signal variation obtained treating PNP_FITC with 15 different *K. pneumoniae* strains in the concentration range of (0–5) × 10^∧^4 bacteria/mL. (D) Statistical analysis showing the robustness of the methods in detecting urease-producing bacteria (ANOVA one-way, *p* = 0.05, Tukey test, *n* = 45).

Once PNP_FITC was fully characterized in its sensitivity and selectivity for urease activity, we tested the probe for the detection of bacteria. As a proof-of-concept, we tested our probe with bacteria isolated from patients hospitalized at a local hospital. Aiming to define the selectivity in detecting bacteria based on their urease activity, PNP_FITC was treated with different bacteria at different concentrations in the range of (0–5) × 10^∧^4 bacteria/mL. The results showed that the proposed direct detection mechanism enabled us to discriminate between urease-producing bacteria such as *K. pneumoniae* and *E. cloacae* from nonurease-producing bacteria, i.e., *P. aeruginosa* and *E. coli* (Figure 5A), with a calculated LOD of 2 × 10^∧^3 and 7 × 10^∧^3 bacteria/mL, respectively, for *K. pneumoniae* and *E. cloacae*. The response of the fluorescent probe when treated with urease was used to calculate the amount of enzyme released by the bacteria, and we found that *K. pneumoniae* and *E. cloacae* produced ca. 2 and 0.2 U/mL at, respectively, 5 × 10^∧^4 and 5 × 10^∧^3 bacteria/mL. The efficacy of PNP_FITC in detecting bacteria via the direct detection of the urease activity was compared with the pH-based method. As shown in Figure 5B, the pH-based method was not sensitive enough to detect the two strains of *K. pneumoniae* (s1 and s2) at the tested concentrations. On the contrary, PNP_FITC was efficient in sensing the bacteria, proving the advantages of the proposed approach. Figure S3A shows the signal variation over time of PNP_FITC when treated with *K. pneumoniae* at 5 × 10^∧^4 bacteria/mL in contrast to the stability of the fluorescent signal observed using the pH-based method. The robustness of our sensing method was tested by treating PNP_FITC with 15 different strains of *K. pneumoniae* isolated from hospitalized patients, each of which was evaluated in triplicates. As shown in Figure 5C, the signal increased when tested with 5 × 10^∧^4 bacteria/mL reaching a maximum of 3-fold increment of the signal with certain strains, indicating a good signal-to-noise ratio. Moreover, statistical analysis (Figures 5D and S3B; *n* = 45) showed that the variation of the fluorescent signal at the different bacterial loads was statistically different down to 5 × 10^∧^2 bacteria/mL, further confirming the robustness and reliability of the proposed method.

The sensitivity of detection achieved with the direct urease probe proposed here was further compared with the fluorescent and colorimetric pH-based methods and alternative approaches reported in the literature. Table 1 summarizes the sensitivity and some relevant features of sensors (i.e., detection time and sensitivity). The LOD for urease obtained with PNP_FITC was comparable with the methods available in the literature, confirming the sensitivity of PNP_FITC. Even though the pH-based detection methods enable the fast detection of urease, they fail in detecting the urease-producing bacteria. Conversely, PNP_FITC proved to be particularly rapid and efficient in detecting urease-producing bacteria as confirmed by the improved LOD and discriminating them from nonurease-producing bacteria. The suitability of PNP_FITC in detecting bacteria can be ascribed to the direct detection of the urease activity, which limits the interference of environmental parameters as is the case for the pH-based approaches, which can be affected by nonurease-related pH changes in the media related to the metabolic activity of bacteria (e.g., lactate production).

**Table 1:** Comparison of PNP_FITC’s Detection Efficacy for Urease and Urease-Producing Bacteria with the Literature

type of sensor	detection method	incubation time	LOD urease	LOD bacteria	ref
fluorescence	pH	30 min	20 μM		(53)
fluorescence	pH	n.a.	0.13 U		(54)
fluorescent/colorimetric	pH	1 h	1.83 U		(55)
colorimetric	pH	n.a.	1.8 U		(56)
potentiometric	pH	1 min	1 U	10^∧^5 CFU/mL	(57)
colorimetric	pH	4 h		n.a.	(11)
colorimetric	NH_3_ gas	11 h		10^∧^8 CFU/mL	(9)
fluorescence	pH	24 h		n.a.	(58)
piezoelectric	NH_3_ gas	8 h		10^∧^8 CFU/mL	(59)
fluorescence	direct detection	5 h	0.4 U/mL	10^∧^3 bacteria/mL	this work

## Conclusions

We propose a fluorescent-based detection method that enables the direct detection of urease. Thanks to its mechanism of detection, the proposed method is not affected by environmental pH, enabling the robust, rapid, and user-friendly detection of urease-producing bacteria with high sensitivity and selectivity. This study confirmed that the fluorescent probe proposed here enables the detection of urease-producing bacteria with improved sensitivity and robustness compared to the standard pH-based approaches. Given the positive results obtained with the strains isolated from human samples, we will further investigate the possibility of exploiting such a sensitive urease probe to discriminate between bacterial infections and noninfectious or viral episodes. The promising results represent a step further toward the ambitious goal of designing multisensing platforms enabling the rapid discrimination among bacteria present in human samples, thus supporting the appropriate diagnosis of infection and enabling prompt administration of the appropriate therapy. Such a screening could ultimately promote the appropriate antibiotic treatment of nosocomial infections. This would have considerable economic benefits for healthcare systems by reducing the length of inpatient stays and limiting the development of antibiotic resistance.

## Supporting Information Available

File reports additional data regarding the characterization of the probe (Figure S1); statistical analysis of the detection efficacy (Figure S2); and additional data regarding the comparison with the pH-based detection method (Figure S3) (PDF)
