# Supplementary material for: Fluorescent Probe for the pH-Independent Rapid and Sensitive Direct Detection of Urease-Producing Bacteria
Source: Anal Chem. 2024 Dec 16;96(52):20578–86. doi: 10.1021/acs.analchem.4c05182 (PMC11696831; doi:10.1021/acs.analchem.4c05182)
Supplement: Supplementary file 1 — ac4c05182_si_001.pdf [file ac4c05182_si_001.pdf]

# Supporting Information

# Fluorescent probe for the pH-independent rapid and sensitive direct detection of urease-producing bacteria

W. C. Albrich[a§], C. R. Kahlert[a,b§], S. Nigg[a], L. F. Boesel[c], G. Giovannini[c*]

a Division of Infectious Diseases, Infection Prevention and Travel Medicine, Kantonsspital St. Gallen, Kantonsspital St. Gallen, Rorschacher Strasse 95, St. Gallen 9007, Switzerland

b Infectious diseases and Hospital epidemiology, Children's Hospital St. Gallen, Claudiusstr. 6, St. Gallen 9006, Switzerland

c Empa, Swiss Federal Laboratories for Materials Science and Technology, Laboratory for Biomimetic Membranes and Textiles, Lerchenfeldstrasse 5, St. Gallen 9014, Switzerland

§ Shared first authors

* Corresponding author: GG giorgia.giovannini@empa.ch

Table of Content

1. Characterization of PNP_FITC ........ S2
2. Statistical analysis of PNP_FITC in detecting urease and selectivity ........ S2
3. Comparison between the direct detection approach (PNP_FITC) and the pH-based method ........ S3

## 1. Characterization of PNP_FITC

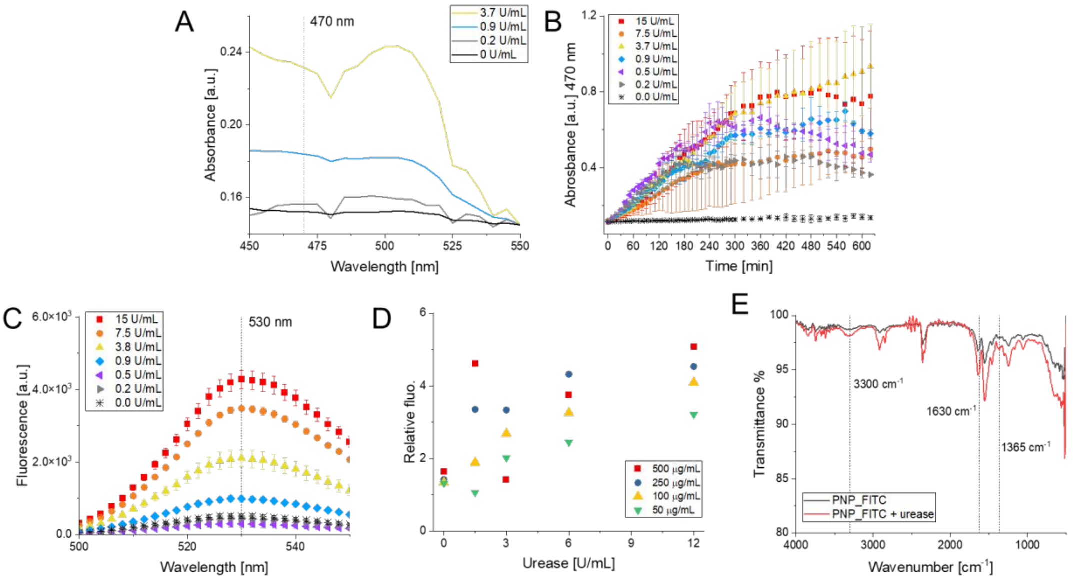


Figure S 1: A) UV-Vis spectrum of PNP_FITC treated with urease (0, 0.2, 0.9, and 3.7 U/mL) showing the increase of the absorbance upon particle degradation (B). 470 nm was selected as the excitation wavelength rather than 530 nm to obtain a larger stock shift between excitation and emission. This limits the possible interference between excitation and emission. C) Fluorescence spectrum recorded after excitation at 470 nm, indicating 530 nm as the emission peak and showing good responsiveness of the probe in response to the enzyme. D) Given the linear response to the urease, 100 μg/mL of PNP_FITC was selected as the concentration for the following assay. E) FT-IR spectra of untreated (black line) and urease-treated (red line) particles (15 U/mL, 60 minutes) showing the increased intensity of the broad peak at 3300 $cm^{-1}$ assigned to the stretching vibration of the ammonia and the strengthening of the peaks at 1630 $cm^{-1}$ and 1365 $cm^{-1}$ indicative for the stretching and vibration of the carbonyl group of bicarbonate, formed as consequence of the dissolution of $CO_2$ in aqueous environment. This chemical analysis confirmed the cleavage of the urea units forming the polymeric matrix of the probe upon enzymatic treatment, leading to the formation of the amino and carbonyl groups.

## 2. Statistical analysis of PNP_FITC in detecting urease and selectivity

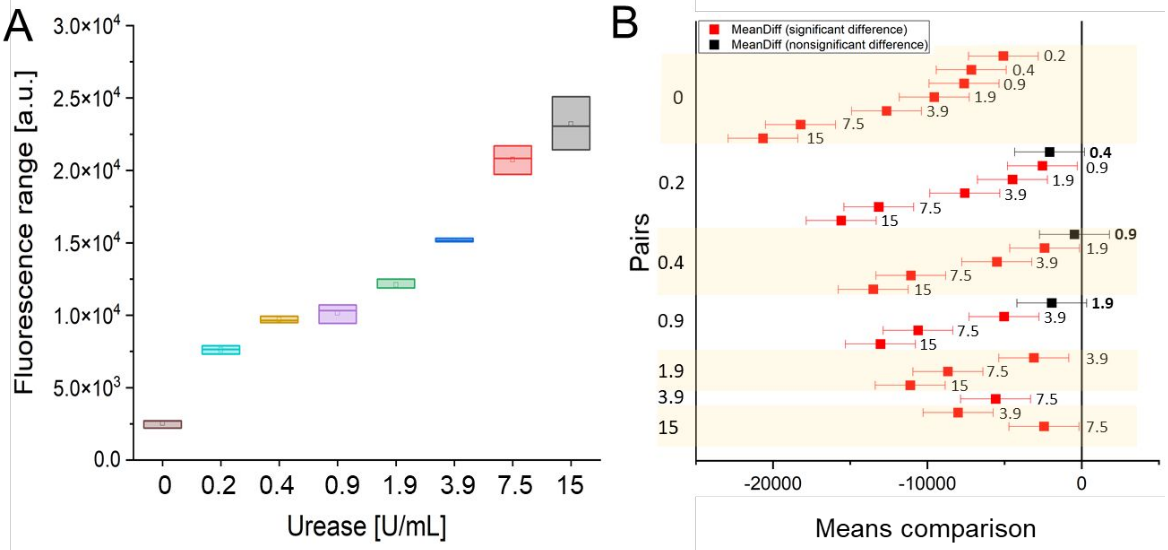


Figure S 2: Graphical representations of the distribution and key statistical measures of the PNP_FITC response to urease. Statistical analysis of the performance of PNP_FITC in detecting urease. A) The box-and-whisker plot distribution of the means

of the signal variation at the different enzyme concentrations. B) Resoluts of Tukey's test showed that the mean values are statistically different down to 1.8 U/mL.

## 3. Comparison between the direct detection approach (PNP_FITC) and the pH-based method

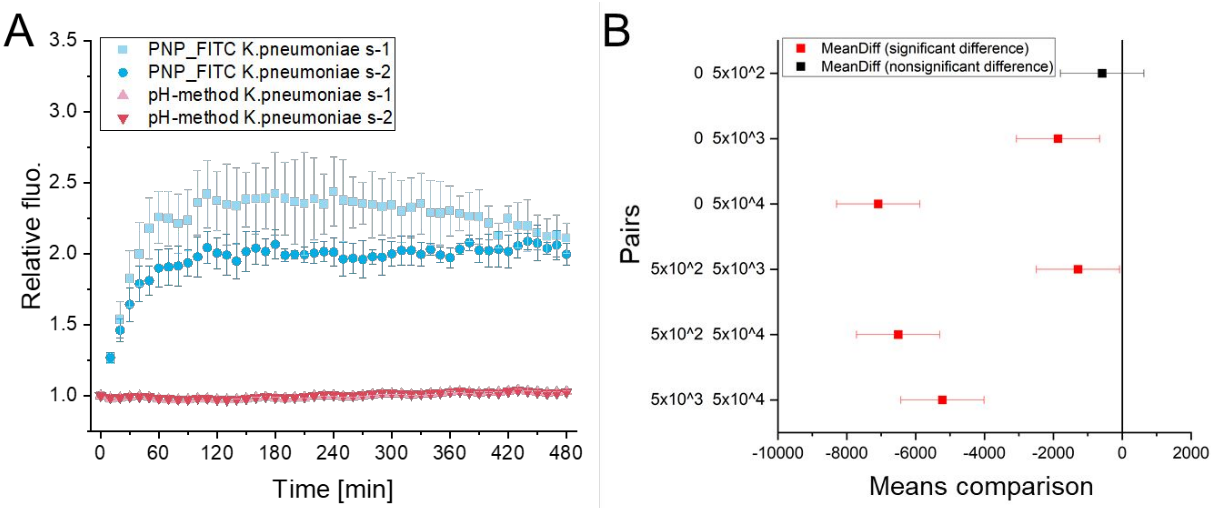


Figure S 3: A) PNP_FITC (blue line) and the pH-based method (red lines) were tested with 5x10^4 bacteria/mL of two *K. pneumoniae* strains (s-1 and s-2). Whereas the PNP_FITC signal increased over time, no changes were detected for the pH-based method, proving the improved detection sensitivity achieved with the here proposed direct probe for urease. B) Tukey's test of ANOVA one-way statistical analysis proves the robustness of the detection method proposed.
